# Supplementary figures and images for: Comparative transcriptomics and phylostratigraphy of Argentine ant odorant receptors
Source: PLoS One. 2024 Sep 3;19(9):e0307604. doi: 10.1371/journal.pone.0307604 (PMC11371221; doi:10.1371/journal.pone.0307604)

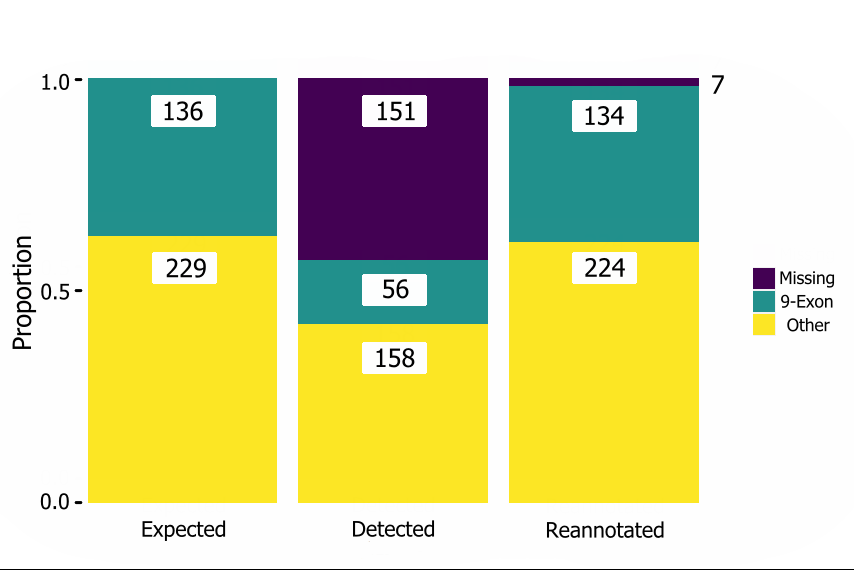

Supplement: S1 Fig — Stacked Barplot indicating the amount of expected odorant receptors (ORs) present in the Argentine ant genome and the ORs that were associated with accession numbers, split between nine-exon ORs and other ORs. Eighty 9-Exon ORs and seventy-four other ORs were unable to be matched to GenBank protein and transcript accession numbers. After OR reannotation, two 9-Exon ORs and five other ORs remain missing from the analysis. (TIF) [file pone.0307604.s001.tif]

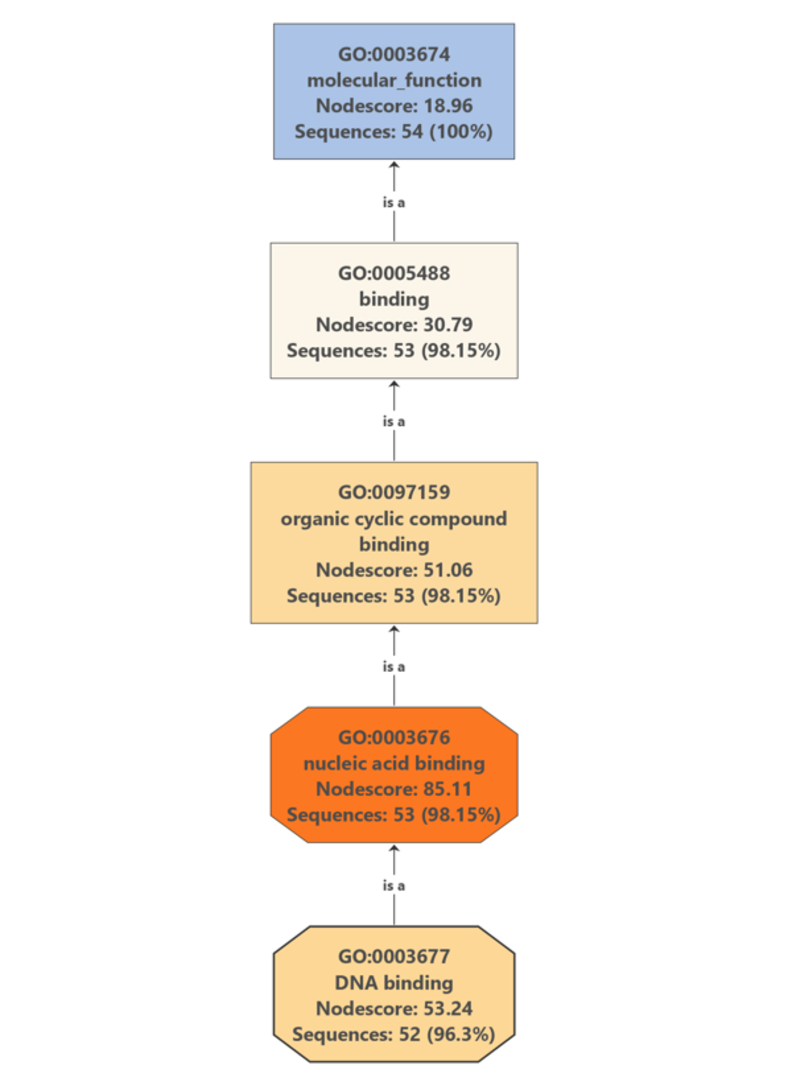

Supplement: S2 Fig — Flow chart generated by BLAST2GO showing GO terms generated from InterProScan of TRPs. Fifty-two of the TRPs show protein signatures associated with DNA binding activity according to InterPro. (TIF) [file pone.0307604.s002.tif]

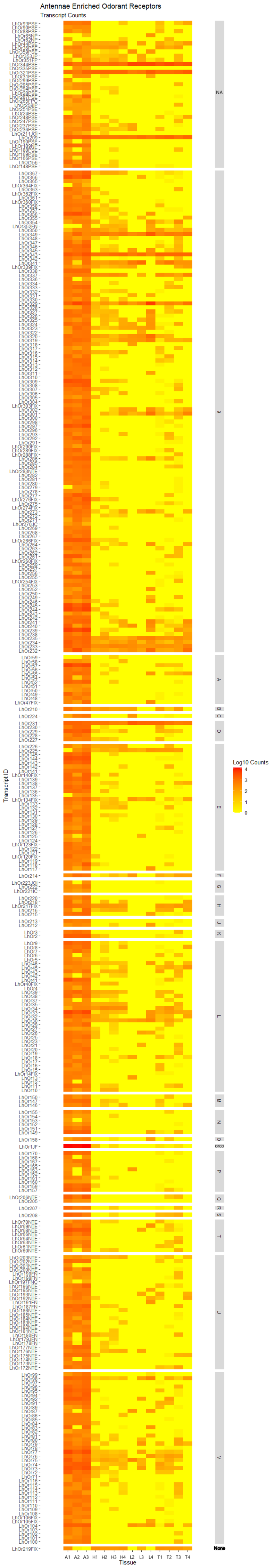

Supplement: S3 Fig — Heat map showing Log10 transcript counts for all L. humile ORs, broken up by subfamily. “NA” ORs were not included in the subfamily analysis conducted in Engsontia et al. 2015. (TIF) [file pone.0307604.s003.tif]
