# Supplementary material for: Comparative transcriptomics and phylostratigraphy of Argentine ant odorant receptors
Source: PLoS One. 2024 Sep 3;19(9):e0307604. doi: 10.1371/journal.pone.0307604 (PMC11371221; doi:10.1371/journal.pone.0307604)
Supplement: Attachment — Submitted filename: review.pdf [file pone.0307604.s009.pdf]

Thank you for your work, it is useful results.

About the method:
How did you handle incomplete gene models (missing regions)?

About the introduction:
"Eusocial insects are some of the most successful insect species on the planet"
In terms of fame I agree, but this seems irrelevant to the biological aspect. You could argue that silverfish are the most successful as they are the oldest class. Most importantly, success could be interpreted to mean fitness success, which is not the point you want to make.

"Altogether, the antennae-upregulated OR genes provide a 234 valuable candidate list to further examine how Argentine ant supercolonies are maintained. 235 Additionally, investigating the function of ORs that are primarily expressed in other tissues can 236 provide new insights on the diversity of function by insect odorant receptors. "
You suggest that it is their perception or lack of it that makes Argentine ants' super-spreaders?
The answer may lie in the ability to perceive and detect, sure, but it can also be in that emission aspect, the label of the entire population may be similar and therefore not trigger aggression, or perhaps the aggression of this species is generally low. You seem to be leaving out alternative hypothesis.

About the result:
You may want to limit the number of figures in your manuscript. You can choose 2 or 3 that support the message you want to send.

The title being about phylostratigraphy, I was expecting a tree somewhere in the "178 3.2 ORs are younger than the rest of the proteome" section.

Fig 2, is there a quicker way to show the benefits of the re annotation? Maybe pick one of the comparisons to present in the manuscript fig, mention the rest and place this figure 2 in supplementary.

Figs 1 to 4 seem redundant, maybe pick one or fusion 2 and it is enough for the story, the rest can go in a supplement.

Fig 5: I don't understand what is C
What is the difference between table 2, 3 and 4 in the supplementary? They seem to be the same things.

Data are missing, you may need to join the read count table and the enrichment output analysis, or a link to it.

About the discussion:
I am not sure I understand your main point. Perhaps it might be interesting to focus on your main result at first with supporting figures and then discuss the interesting parallel element in relation to the supplementary.

If the point is:
- re-annotate the OR families/9-exon
- clustering/estimate their emergence through the evolutionary history of insects
-> It might be useful to emphasise the importance of the intron phase, OR properties (etc.) in OR categorisation rather than just in the putative ecological context. Present fewer figure and discuss it under a broader paradigm than recognition and more functional/proximal.

If the point is to look at how OR distribution in this species affects recognition capabilities and explains invasive spread, then make it clearer. But I don't think this study answers that question directly so it would be care full to present it as a hypothesis rather than a result.

Also, this more of an opinion than a necessary review point, 9-exons are probably not the only OR family that plays a role in recognition. They bind CHCs, that is undeniable. CHCs probably play an important role in recognition, sure. But nothing so far has proved that they are the only cues, they are present in huge quantities on the cuticle, and the 9-exon family is largely represented in eusocial insects, which seem to be the ones that need the most accurate recognition. But quantity is not always the strongest argument and nothing tell us that they are the only family candidate. On all human muscles, only a few are related to express emotion, they evolve a parallel function that mobility, but other channel are used (tears, speech, etc.) likewise CHC are initials use to fight against desiccation, some might have evolved and other component probably play a role in recognition.
You don’t seem to disagreeing with this point but all your proposition for further investigation seems to restrict to this family.

About the format:
Line 104 and other “Two hundred and fourteen out of 365 OR” Make it easier to read and select numbers consistently throughout the manuscript.

On line 164 you introduce 9E without explaining the abbreviation. Make sure they are all spelt out before you use them.
