# Supplementary material for: Comparative transcriptomics and phylostratigraphy of Argentine ant odorant receptors
Source: PLoS One. 2024 Sep 3;19(9):e0307604. doi: 10.1371/journal.pone.0307604 (PMC11371221; doi:10.1371/journal.pone.0307604)
Supplement: Attachment — Submitted filename: Response to Reviewers.docx [file pone.0307604.s010.docx]

Reviewer 1:

1. I am not sure I understand your main point. Perhaps it might be interesting to focus on your main result at first with supporting figures and then discuss the interesting parallel element in relation to the supplementary.
If the point is:
- re-annotate the OR families/9-exon
- clustering/estimate their emergence through the evolutionary history of insects
-> It might be useful to emphasise the importance of the intron phase, OR properties (etc.) in OR categorisation rather than just in the putative ecological context. Present fewer figure and discuss it under a broader paradigm than recognition and more functional/proximal.

If the point is to look at how OR distribution in this species affects recognition capabilities and explains invasive spread, then make it clearer. But I don't think this study answers that question directly so it would be care full to present it as a hypothesis rather than a result.

**Response: The intention of this study is to identify ORs that are likely involved in nestmate recognition by determining which nine-exon ORs are expressed primarily in the antennae. There is very little known about the role of ORs in nestmate recognition, only that nine-exon ORs are likely involved in the process. Our results show that the expression of nine-exon ORs varies across tissue, and only a subset that are selectively upregulated in the antennae are likely involved in nestmate recognition. This enables further investigation of these specific ORs to determine how they function within nestmate recognition. We have added a clarification to lines 53-59.**

3. How did you handle incomplete gene models (missing regions)? What is the difference between table 2, 3 and 4 in the supplementary? They seem to be the same things. Data are missing, you may need to join the read count table and the enrichment output analysis, or a link to it.

**Response: Gene models derived from protein sequence data from Smith et al. 2011 were kept as-is, we have clarified this in lines 127-128. Captions for Tables S2-5 were clarified to explain the contents of each table. Proteins with no associated data in these tables are the proteins that do not have NCBI annotations.**

Reviewer 2:

The transcriptomic dataset needs to be analyzed further to check with ORs are specifically upregulated in different tissues.

**Response: We have included this analysis as Figure 3, and in lines 167-168.**

I would further recommend performing phylogenetic analyses on the ORs to investigate which subfamilies of ORs are present in antennae and other tissues. I would expect that the 9-exon subfamily to be enriched in the antennal dataset and other subfamilies to be expressed in non-antennal tissues. Additionally, the authors could extend the phylogenetic analyses with the ORs to other species of ants with well-annotated transcriptomes and this would help to identify whether there are any specific subfamilies of ORs that have undergone species-specific expansion.

**Response: While extending the phylogenetic investigation of ant ORs is an important area of research, investigating expansion of OR subfamilies across Formicidae species has already been conducted in Engsontia et al. 2015. However, the suggestion of adding the *L. humile* subfamily data into our OR expression data was valuable, and we have incorporated these into Figure S3.**

This could further be expanded to compare ORs in native and introduced regions to identify the ORs that might play a role in reduced aggression in the latter type.

**Response: While investigating the differences underlying native and introduced populations of *L. humile* is vital to understanding how invasive supercolonies are formed and maintained, we believe that comparing ORs between native and introduced subfamilies will not be fruitful, given the relatively recent population separation in evolutionary time.**

Another potential analysis could be to look for genomic tandem arrays in the ORs and add a section on what proportion of ORs are present in tandem arrays.

**Response: We have included an analysis of ORs in tandem arrays as Figure 6, and in lines 170-174.**

I do realize that long-read RNA-Seq data is a requirement for this kind of analysis, and the authors might not have that available as of now. However, I would recommend collecting long-read data and adding to this manuscript since it would improve the power of the analyses as well as the quality of the manuscript.

**Response: While long-read sequencing can be valuable for investigating genomic structure, we are not able to perform long-read sequencing at this time, though it would be a fruitful avenue for future work in this area.**

Lines 82-86: How many colonies were used to collect the workers? Were workers from different colonies treated as separate biological replicates? How many biological replicates were collected for each body part? Figure 1 shows that head and thorax have 4 replicates each, and antennae and legs have 3 replicates each. Please explain the removal of replicates for antennae and legs. Were heads and thoraces of these workers used in the analyses?

**Response: Added lines 91-92 to explain the loss of antennal and leg samples. Head and thoracic tissues were still used for transcriptomic analysis.**

Why were the abdomen not included in the data collection? Is it definitely known that ORs are not expressed in abdomens of ants?

**Response: Abdomen data were not collected in this analysis due to concerns that enzymes contained in the abdomen would degrade mRNA and render any transcriptomic analysis poor-quality and wasteful.**

Lines 96-97: Were the transcript counts normalized? Please add this detail.

**Response: DESeq2 uses un-normalized count data as input. This information was added in line 107.**

Line 107: Were the ORs annotated in a previous study? If so, please cite the appropriate article.

**Response: OR annotations were obtained from *L. humile* draft genome paper. This paper is on line 125 in our manuscript.**

Lines 130-131: Please expand on this analysis for calculating node distance. How was the node information used in downstream analyses. Phylostratigraphy is one of the major analyses in this study and requires expansion in the methods.
A general recommendation would be to submit the analyses codes.

**Response: Explanation of node distance calculation was added in lines 145-146.**

Lines 138-139: “We identified 4288 antennae-upregulated genes …”. Figure 2A only shows a total of 4280 antennae upregulated genes. Is this a calculation error? Are 8 genes not shown in the plot? If so, please explain why those 8 genes were dropped.

**Response: This was a typographical error that has been corrected.**

Lines 140-144: “… representing one third of the detected ORs.” Are these “detected ORs” based on the total ORs in Figure S1? It would be nice to either mention the total “detected ORs” in these two cases or refer to Figure S1.

**Response: References to Figure S1 were added to the relevant lines (157-165).**

Lines 191-194: It is not clear what the authors mean by “after adding rest of the OR proteins”. Please explain.

**Response: Line 221 was rewritten to clarify that the analysis was run on reannotated ORs.**

Lines 204-206: “… odorant receptor genes do not appear to show any changes …”
It is not clear which contrasts are the authors referring to here. It sounds as if the authors are comparing ORs between lab and field colonies but not stating it clearly. Please mention if such a comparison was done. If so then the statement, “ …. so the OR findings discussed should be applicable to field colonies” is misleading.

**Response: Our point is that prior literature indicates that some genes change their expression between lab and field colonies of *L. humile*, and it’s necessary to verify whether or not ORs exhibit this behavior before our results can be extrapolated onto field conditions. We have moved this section to lines 251-257 and clarified our intentions.**

Figure 3: Were the p-adjusted values used for this volcano plot?

**Response: Updated Fig. 3 to clarify adj. p-value was used for volcano plot**

Figure 5: Please expand on how these
I would recommend adding “All Genes” column to Figure 5B.

**Response: We have included All Antenna-Upregulated Transcripts as Figure 5A.**

Lines 77, 157, 167: Italicize the species name.

**Response: Corrected.**

Line 229 and Figure 5A: Please stick to either TRG or TRP.

**Response: Corrected mentions of “TRG” to “TRP”.**
