# Supplementary material for: Comparative transcriptomics and phylostratigraphy of Argentine ant odorant receptors
Source: PLoS One. 2024 Sep 3;19(9):e0307604. doi: 10.1371/journal.pone.0307604 (PMC11371221; doi:10.1371/journal.pone.0307604)
Supplement: Attachment — Submitted filename: Response to Reviewers - 6.29.24.docx [file pone.0307604.s011.docx]

Editor:

**The reviewers were happy with the revised version and I like it as well. I have one minor thing that I didn't catch before: I initially stumble when people show me upset plots because they appear to lack a description of what's on the x-axis (I know it's there but it's odd not so see words), and some readers may have the same problem. It would be nice if you could add 1-2 sentences to the figure legend explaining how to read the plot, i.e. what all the bars (also the horizontal ones in the bottom left) show (upregulated in the antenna vs. all those tissues marked with a dot), and what test that was exactly (differentially expressed in antenna vs. pooled (leg, thorax, head) OR DE in all three comparisons A vs L, A vs T, A vs H separately).**

Response: We have updated captions for Figures 2 and 3 to include an explanation of how to read the upset plots.
